# 

**Published:** 1883-10

**Authors:** 


SURG GEN'L'S OFFS

Vol. XX. October, 1883. No. 3.

### The Bistoury.

Thad S. Up de Graff, M.D.
EDITOR.

ELMIRA, N.Y.

